# Supplementary material for: Retinal Neurodegeneration in an Intraocular Pressure Fluctuation Rat Model
Source: Int J Mol Sci. 2024 Mar 26;25(7):3689. doi: 10.3390/ijms25073689 (PMC11011540; doi:10.3390/ijms25073689)
Supplement: Supplementary file 1 [file ijms-25-03689-s001.zip › ijms-2899465-supplementary.pdf]

Supplemental Data

Retinal Neurodegeneration in an Intraocular Pressure Fluctuation Rat Model

Jeong-Sun Han, Chan Kee Park, Kyoung In Jung

Department of Ophthalmology, Seoul St. Mary's Hospital, College of Medicine, The Catholic University of Korea, Seoul, Korea

*Kyoung In Jung

Email: ezilean@hanmail.net

This Word file includes:

Figures S1 to S5

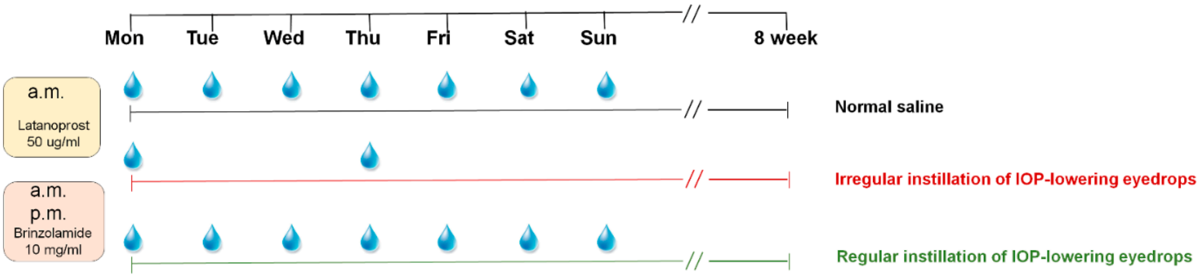

Figure S1. The time schedule of the experiments. Drug treatment assigned to the irregular instillation group, regular instillation group, or control group. Intraocular pressure (IOP)-lowering eyedrops (latanoprost and brinzolamide) were administered to rats on Monday and Thursday in the irregular instillation group and daily in the regular instillation group for 8 weeks. Meanwhile, saline was given to the control group daily for 8 weeks.

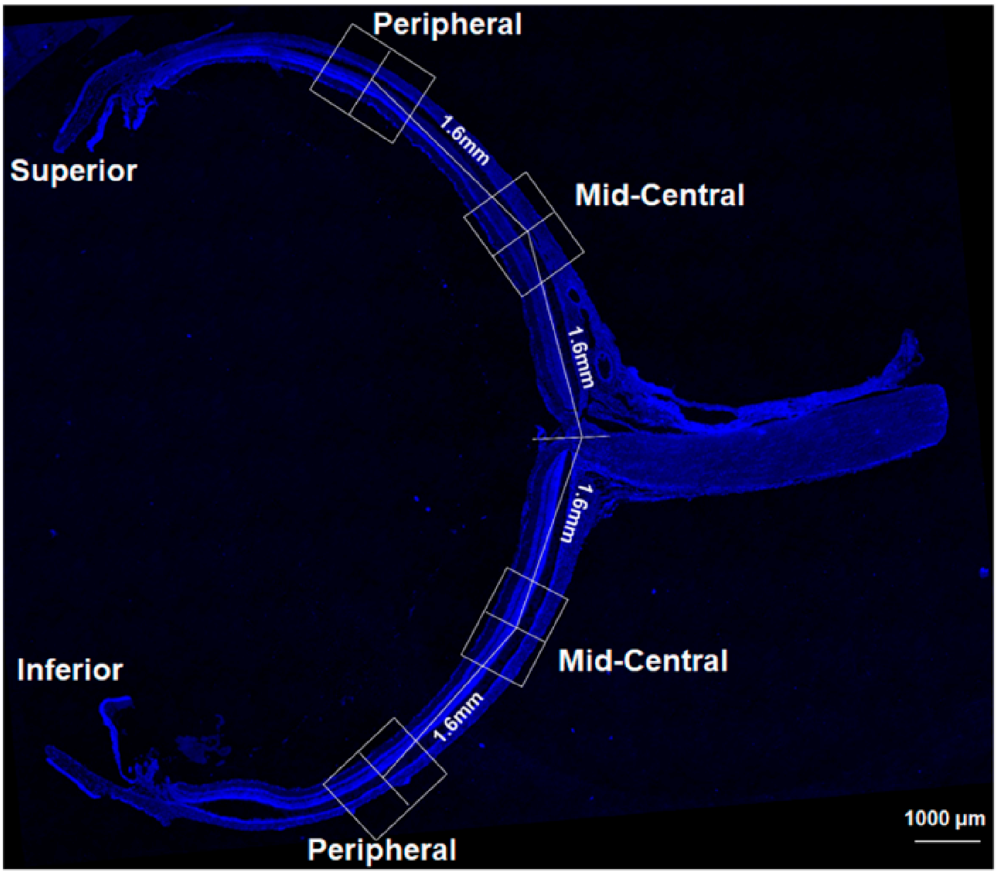

Figure S2. Representative posterior eyecup image stained with DAPI for analysis. Each eyecup was divided into 2 mid central (1.5 mm from the optic nerve), 2 peripheral areas (3.5 mm from the optic nerve) for image analysis.

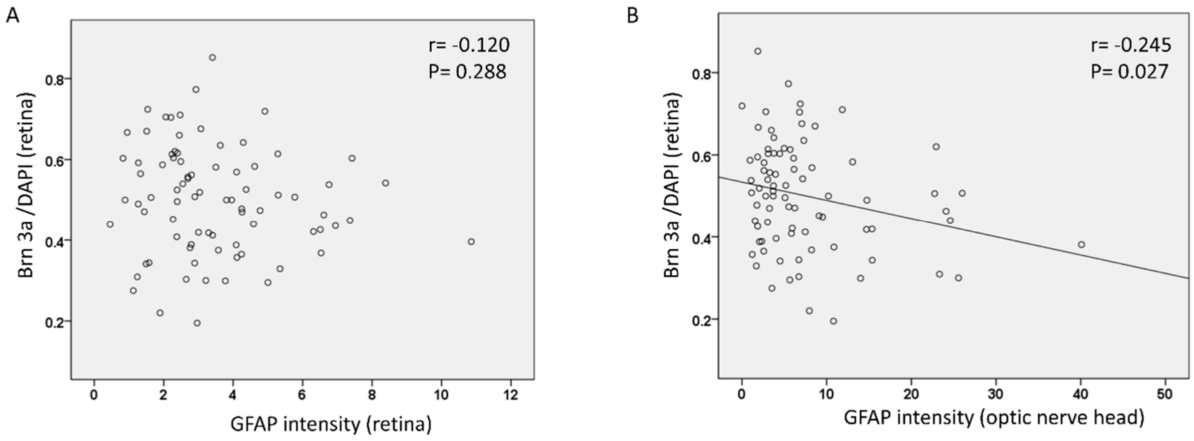

Figure S3. The correlation between gliosis and the degeneration of retinal ganglion cells (RGCs). (A) There was no significant difference between the intensity of glial fibrillary acidic protein (GFAP) expression and the proportion of cells positively stained with Brn3a per DAPI (r = −0.120, *P* = 0.288). (B) GFAP intensity in the optic nerve head was negatively correlated with the proportion of cells positively labeled with Brn3a per DAPI in the retina (r = −0.245, *P* = 0.027).

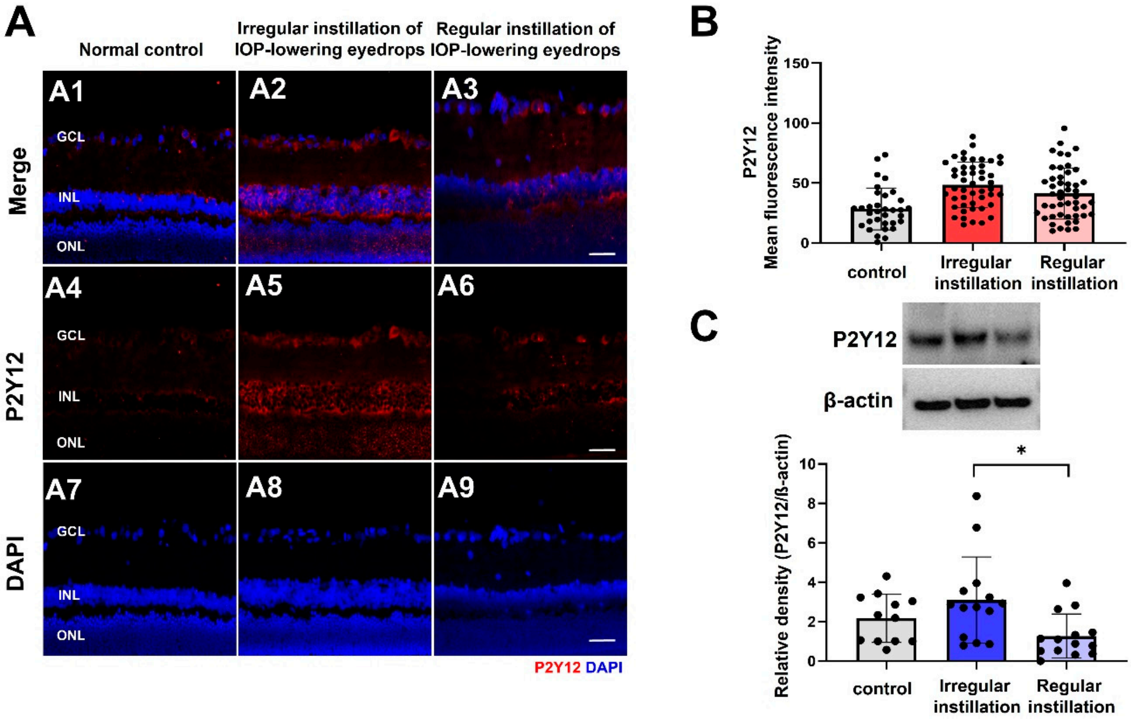

Figure S4. P2Y21 expression. (A, B) Immunofluorescence staining of P2Y12 was increased in the ganglion cell layer (GCL) and inner nuclear layer in the irregular instillation group. (C) Expression of P2Y12 by western blotting was upregulated in the irregular instillation group, but it did not differ significantly from that in the normal control group (**p* = 0.05). Scale bar = 20 μm.

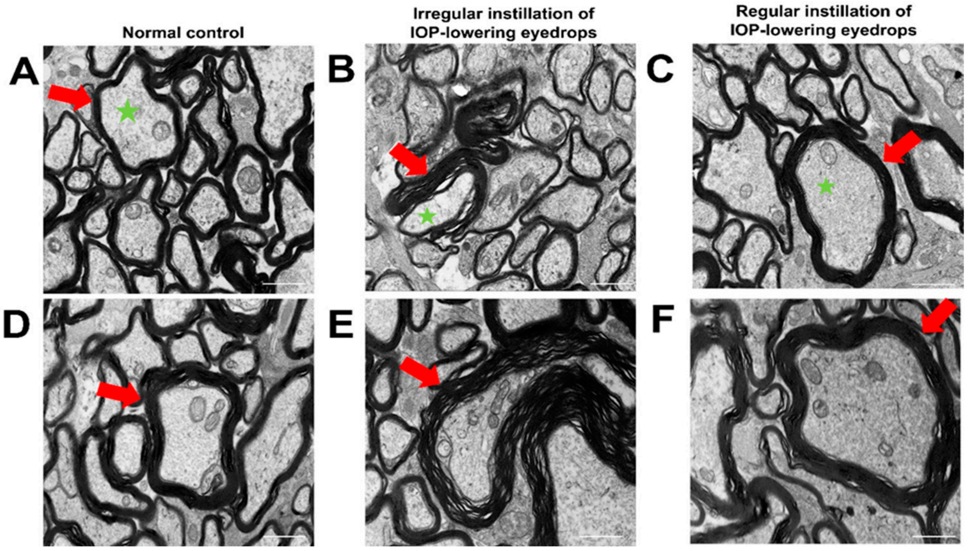

Figure S5. Ultrastructural findings of the optic nerve. Electron microscopy of cross-sections of the myelinated optic nerve region. In the normal control group (A, D), normal axoplasm surrounded by well-organized myelin sheathing was found. In the irregular instillation group, some of the myelin sheath (arrow) was disintegrated and some of the axoplasm was relatively sparse (asterisk), even though many axons retained a normal appearance (B, E). The myelin sheath and axoplasm were relatively preserved in the regular instillation group (C, F). Scale bar=1 μm
